# Factors contributing to visual intrapartum cardiotocograph interpretation variation among healthcare professionals: An integrative review

**DOI:** 10.1371/journal.pone.0315761

**Published:** 2025-01-24

**Authors:** Sanele Lukhele, Fhumulani Mavis Mulaudzi, Rodwell Gundo

**Affiliations:** Faculty of Health Sciences, Department of Nursing Sciences, University of Pretoria, Pretoria, South Africa; University of Saskatchewan, CANADA

## Abstract

The reliability of cardiotocographs as diagnostic tools for fetal well-being is hampered by interpretational variations among healthcare professionals, contributing to high rates of cesarean sections and instrumental deliveries. While adjunct technologies may be used to confirm cases of fetal distress, those in resource constrained areas continue to rely on visual cardiotocograph interpretation to come up with the diagnosis of fetal hypoxia. This study investigated the factors contributing to variations in the visual interpretation of intrapartum cardiotocograph among healthcare professionals in the absence of adjunctive technologies. In this integrative literature review, we conducted a literature search of the following electronic databases: EBScohost, PubMed, Web of Science, and Scopus. The following search terms and Boolean operators were used: (Intrapartum OR Labor OR Labour OR Childbirth OR Birth OR Delivery) AND (Cardiotocography OR CTG OR "Electronic Fetal Monitoring" OR EFM) AND (Interpretation OR Analysis) AND (Variations OR Differences) AND (“Healthcare Workers” OR Nurses OR “Medical Workers” OR “Healthcare Professionals” OR Midwives OR Obstetricians). After removal of duplicates, a total of 1481 articles and titles were screened, 60 full-text articles were examined to verify whether they addressed the scope of the literature review. Nine articles addressed the factors contributing to variations in the visual interpretation of intrapartum cardiotocographs among healthcare professionals. The quality of the studies was appraised using the Quality Appraisal Tool for Studies of Diagnostic Reliability. Thematic analysis identified the following themes: 1) Interpretational variations in cardiotocograph characteristics among health professionals, and 2) factors leading to increased interpretational variation among healthcare professionals. Our results highlight the need for increased cardiotocograph training to improve consistency among health professionals, especially for suspicious and pathological traces, which often lead to cesarean section.

## Introduction

Cardiotocographs are used to monitor fetal well-being during labor and were introduced approximately 50 years ago [1]. The cardiotocograph is a part of routine intrapartum care monitoring in middle-income [2] and high-income countries [1]. Cardiotocograph monitoring and interpretation aims to prevent intrauterine fetal death by confirming fetal well-being. Unfortunately, cardiotocography has faced significant scrutiny over the years due to inconsistencies in interpretation. The interpretation of tracings varies among professionals, and the same practitioner may interpret the same tracing differently at different times [3]. This may lead to inappropriate intrapartum interventions [4]. Adding to the ongoing discourse on cardiotocograph monitoring, research has demonstrated its association with an increased incidence of cesarean sections and instrumental deliveries [5].

Intrapartum cardiotocographs are interpreted according to clinical practice guidelines. Healthcare professionals in different parts of the world use specific official guidelines to interpret cardiotocographs. There are approximately 13–17 cardiotocograph interpretation guidelines available worldwide [6–8]. Among these guidelines, the International Federation of Obstetrics and Gynecology (FIGO) guidelines [9], the American College of Obstetricians and Gynecologists (ACOG) guidelines [10], and the National Institute for Health and Care Excellence (NICE) guidelines [11] are the most commonly used [12]. These guidelines form part of the baseline knowledge that healthcare professionals should possess when interpreting cardiotocographs.

A normal cardiotocograph comprises a stable baseline fetal heart rate (FHR) of 110–160 bpm without significant decelerations. Normal variability of 5–25 beats per minute (bpm) and alternate periods of increased variability with or without acceleration is called cycling behavior and is a vital behavioral state of normal-term or near-term fetuses [13]. Anything outside these parameters is considered abnormal and warrants further consultation and interventions. Cardiotocograph surveillance enables healthcare professionals to study different FHR patterns and uterine contractions during the intrapartum period. These patterns indicate the fetal state and adaptation to the stress of labor. Unfortunately, fetal heart rate patterns can be very complex, which may contribute to a lack of standard interpretation among healthcare professionals [4]. Previous studies have identified the following factors contributing to interpretational variation among healthcare professionals: years of experience [14], paper speed settings [15], and the use of four-tier ST segment analysis (STAN) guidelines, with the availability of electrocardiographic ST waveform data [16].

Visual interpretation of cardiotocographs and fetal heart rate monitoring through a handheld Doppler device and the Pinard fetal stethoscope remain the primary methods of monitoring fetal well-being during labor. Additional tests used in low- and middle-income countries include fetal acoustic stimulation, fetal scalp stimulation tests, and fetal scalp oximetry [2]. High-income countries have attempted to improve cardiotocograph interpretation by introducing fetal blood sampling and STAN of the fetal electrocardiogram [17,18]. Fetal blood sampling has been discontinued in countries with a high burden of the Human Immunodeficiency Virus (HIV), such as South Africa, as part of the prevention of mother-to-child transmission strategy [19]. Additionally, fetal electrocardiograms cannot be performed in these countries because they require a fetal scalp electrode, which is an invasive procedure and contributes to the vertical transmission of HIV.

Recently, a systematic review summarized and assessed the existing inter- and intraobserver reliability research on intrapartum FHR interpretation [12]. This review included studies where fetal monitoring was performed via cardiotocography and intermittent auscultation. The findings of this review revealed substantially more intraobserver agreement than interobserver agreement. Furthermore, clinicians have demonstrated greater reliability and agreement for basic fetal heart rate features than for overall classification [6]. Another systematic review compared visual and computerized cardiotocograph interpretations to determine whether automated cardiotocograph interpretation resulted in improved perinatal outcomes [20]. These reviews, however, did not identify the factors contributing to inter- or intraobserver variability during FHR interpretation. Therefore, this literature review assessed the factors contributing to variations in visual intrapartum cardiotocograph interpretation among healthcare professionals.

## Review question

What are the factors contributing to intrapartum cardiotocograph interpretation variation among healthcare professionals?

## Methods

### Design

An integrative literature review followed the Preferred Reporting Items for Systematic reviews and Meta-Analyses (PRISMA) guidelines and was guided by Whittemore and Knalf’s five stages: problem identification, literature search, data evaluation, data analysis, and the presentation of findings [21].

### Eligibility criteria

The inclusion criteria for the studies were as follows: 1) focused on interpretation variation of visual intrapartum cardiotocograph interpretation; 2) had to be healthcare professionals of any category who interpreted cardiotocographs during the intrapartum period; 3) had to have complete reports with full text available; and 4) had to be reported in the English language.

Exclusion criteria for the literature review were: 1) studies that focused on intrapartum cardiotocograph interpretation among healthcare professionals with the aid of adjunctive technologies, and 2) letters and commentaries were excluded because the review focused on empirical research studies.

### Information and search strategy

A literature search was conducted from January 15, 2024 to February 5, 2024. A second confirmatory search was conducted on the July 3, 2024 with the assistance of a librarian. The following databases were searched: EBScohost, PubMed, Web of Science, and Scopus. The reference lists of studies that were included in the study were also searched. The following search terms were used: (Intrapartum OR Labor OR Labour OR Childbirth OR Birth OR Delivery) AND (Cardiotocography OR CTG OR "Electronic Fetal Monitoring" OR EFM) AND (Interpretation OR Analysis) AND (Variations OR Differences) AND (“Healthcare Workers” OR Nurses OR “Medical Workers” OR “Healthcare Professionals” OR Midwives OR Obstetricians). The search was expanded to include similar terms and concepts such as medical workers.

### Data extraction

**Fig 1:**
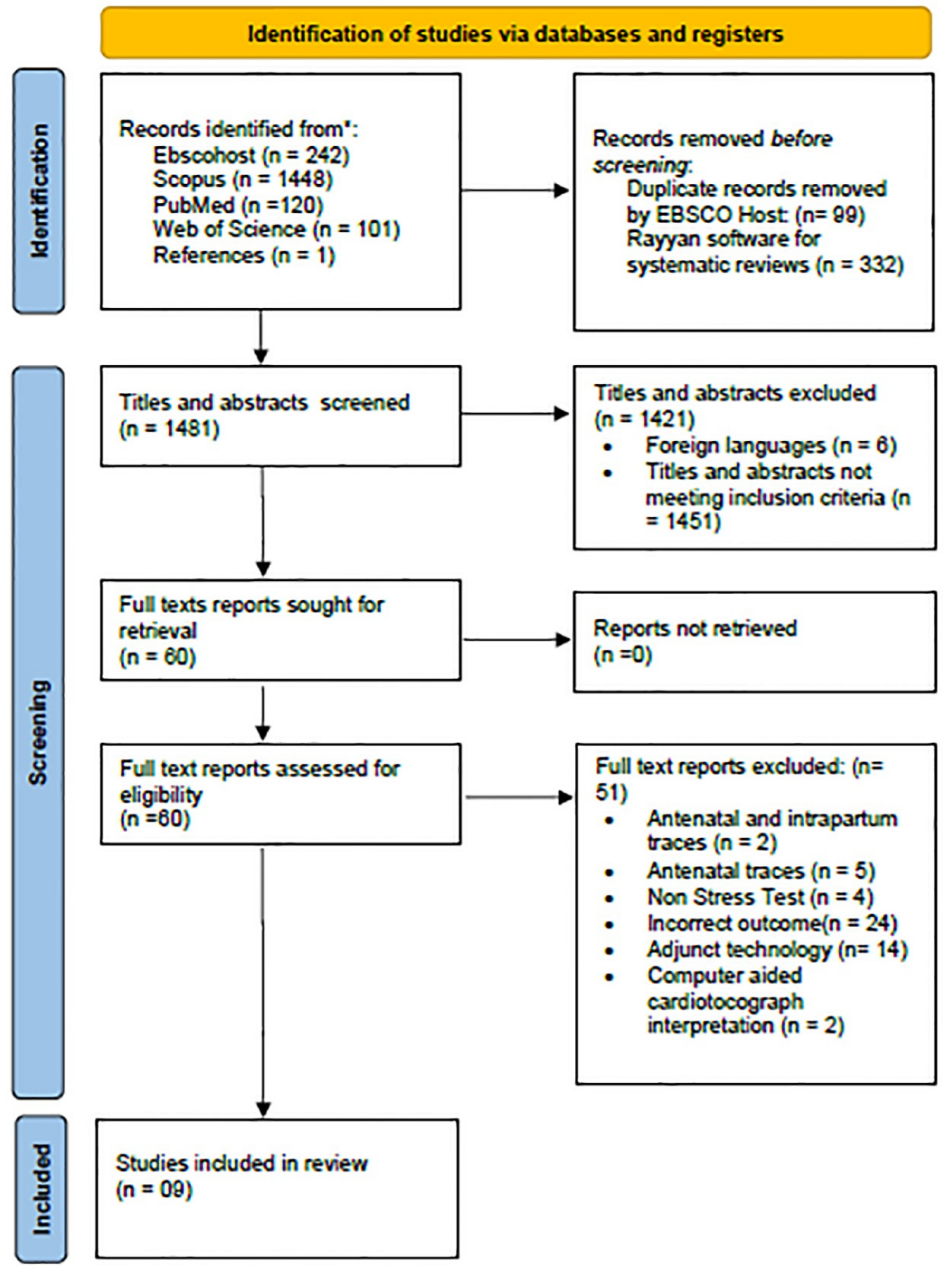
PRISMA flow diagram of the literature review search process. 1. Page MJ, McKenzie JE, Bossuyt PM, Boutron I, Hoffmann TC, Mulrow CD, et al. The PRISMA 2020 statement: an updated guideline for reporting systematic reviews. International journal of surgery. 2021;88:105906.

The first author and a librarian conducted the literature search. A total of 1912 results were identified during the database search. EBSCO Host automatically removed 99 duplicates during extraction. A total of 1813 titles were imported into EndNote 21 reference management software. Thereafter, the results were exported into Rayyan Software for Systematic Reviews [22]. A total of 332 duplicate studies were detected and removed. The total numbers of remaining titles and abstracts was therefore 1481. The remaining titles and abstracts were then dually screened in accordance with the inclusion and exclusion criteria. A total of 60 full-text articles were then screened for eligibility. One further result was identified from the citation search during the full-text screening of the included studies. Only nine studies were included in the review. This process is presented in the PRISMA flow diagram in Fig 1.

### Data analysis

The data were analyzed according to Braun and Clarke’s [23] six phases of thematic analysis. The researchers first familiarized themselves with the data from the included articles, by reading and rereading the articles. In the second phase, initial codes were generated. The third phase involved organizing similar codes into potential themes. In the fourth phase, the themes were analyzed to ascertain their fit into the entire dataset. Phase five involved naming and defining the themes. In phase six, the researchers reported on the themes in this article.

### Quality assessment of studies included in the review

The quality of the included studies was assessed using the Quality Appraisal Tool for Studies of Diagnostic Reliability (QAREL) [24]. This is an eleven-item scale used to assess studies reporting diagnostic reliability. The agreement of two or more observations using the same medium is called diagnostic reliability [24]. Table 1 below is an overview of the QAREL quality appraisal of the studies included in the review.

**Table 1:** QAREL quality appraisal.

	Item 1	Item 2	Item 3	Item 4	Item 5	Item 6	Item 7	Item 8	Item 9	Item 10	Item 11	Score
Blix et al.,	Yes	Yes	No: midwives and obstetricians Yes: experts	N/A	No: midwives and obstetricians Yes: experts	No: midwives and obstetricians Yes: experts	No: midwives and obstetricians Yes: experts	No: midwives and obstetricians N/A for the experts	N/A	Yes	Yes	4/11 for midwives and obstetricians; 8/11 for experts
Devane and Lalor.,	Yes	Yes	Yes	No	Yes	Yes	Yes	Unclear	Unclear	Yes	Yes	8/11
Palomaki, et al.,	Yes	Yes	Yes	N/A	Yes	Yes	Yes	N/A	N/A	Yes	Yes	8/11
Gyllencreutz, et al.,	Yes	Yes	Yes	Unclear	Yes	Yes	Yes	Yes	Yes	Yes	Yes	10/11
Ghi, et al.,	Yes	Yes	Yes	N/A	Yes	Yes	Yes	N/A	N/A	Yes	Yes	8/11
Santo et al.,	Yes	Yes	Yes	N/A	Yes	Yes	Yes	N/A	N/A	Yes	Yes	8/11
Pruksanusak et al,.	Yes	Yes	Yes	Yes	No	Yes	Yes	Unclear	Yes	Yes	Yes	9/11
Amadori et al.,	Yes	Yes	Yes	No	Yes	Yes	Yes	Unclear	Yes	Yes	Yes	9/11
Li et al.,	Yes	Yes	Yes	Yes	Yes	Yes	Yes	N/A	N/A	Yes	Yes	9/11

Item 1: Was the test evaluated in a representative sample of those to whom the authors intended the results to be applied? Item 2: Was the test performed by observers representative of those to whom the authors intended the results to be applied? Item 3: Were the observers blinded to the findings of the other observers during the study? Item 4: Were the observers blinded to their own prior findings on the test under evaluation? Item 5: Were observers blinded to the subjects’ disease status or the results of the accepted reference standard for the target disorder (or variable) being evaluated? Item 6: Were observers blinded to clinical information that was not intended to form part of the study design or testing procedure? Item 7: Were observers blinded to additional cues that are not part of the test? Item 8: Was the order of examination varied? Item 9: Was the stability (or theoretical stability) of the variable being measured taken into account when determining the suitability of the time interval among repeated measures? Item 10: Was the test applied correctly and interpreted appropriately? Item 11: Were appropriate statistical measures of agreement used?

### Ethical considerations

This review was undertaken as part of a larger study for doctoral qualification purposes. The study was approved by the University of Pretoria Faculty of Health Sciences Ethics Committee (Reference No.: 580/2021, approved 10/28/2021).

## Results

### Description of included studies

The researchers selected 60 articles for full-text review. Of those, nine met the inclusion criteria for the integrative review. The studies included in this review used quantitative designs and were conducted in Norway [25], Ireland [3], Finland [26], Sweden [27], Portugal, Boston and London [28], Thailand [29], Italy [30,31], and China [32]. The studies used methods such as asking observers to interpret the same traces using two different FHR classification systems [29] and asking observers to interpret various types of cardiotocograph traces using a single FHR interpretation classification system [25,26,28,32]. Four of the studies reported intra- and interobserver agreement [3,27,29,30], while the other five reported interobserver agreement [25,26,28,31,32].

#### Statistical analysis measures for intrapartum cardiotocograph interpretational variation

The studies included in this review used different statistical methods to assess reliability and agreement. Reliability and agreement are two separate concepts that describe how a particular instrument detects real variability between assessors, and whether repeated measures of the instrument yield consistent results [33]. Agreement shows the exact magnitude of how close test scores for repeated measures are [33]. Reliability is defined as the proportion of variation between scores of the same subjects by different observers, which may even be different from the total variability of all scores in the sample [34]. The kappa (*κ*) statistic provides a numerical rating of the difference between inter- and intraobserver agreement, which is actually present, and that which is expected to be present by chance alone. The kappa scale ranges from -1 to 1, where 0 represented exact agreement by chance, 1 represented perfect agreement, and negative values represented agreement less than chance [35]. The weighted kappa (κw) reflects the degree of disagreement, so that greater emphasis is placed on large differences between ratings and not small ones [35].

The proportions of positive agreement and specific agreements depict the overall agreement between observers for each category under investigation [33]. The result is often expressed as a percentage; for example, 0.85 indicates 85% agreement between observers [36]. The main outcome measurements of each study are shown in Table 2.

**Table 2:** Overview of studies included in the review.

	Authors, (year), country	Study objective	Study design	Subjects	Observers (number, degree and/or expertise)	Main outcome measure	Main results
1.	Blix, Sviggum, Koss, Pal Oian, (2003) Norway [25]	Agreement in CTG interpretation between midwives and obstetricians in a clinical setting and two experts in a nonclinical setting.	Quantitative, observational study. Admission CTGs were analyzed, interpreted and classified.	845 CTGs from both low and high-risk women.	Practicing midwives and obstetricians. Two experts who were not in practice.	Reliability- weighted kappa (κw) Agreement- Proportional agreement (Pa)	Interobserver agreement: κw = 0.38, between expert 1 and expert 2.
2.	Devane and Lalor, (2005) Ireland [3]	Inter and intraobserver agreement in midwives’ visual interpretation CTGs	Randomized trial. Participants visually interpreted three CTG tracings on two occasions, two hours apart.	Three tracings reproduced after permission granted from original publisher.	28 Midwives. 2–21 years’ experience	Agreement- Cohen’s kappa (*κ*)	Overall intraobserver agreement was fair to good (*κ* = 0.48) to excellent (*κ* = 0.92). Observer’s classifications changed in 18% of normal traces, 29% in suspicious cases and 11% in pathological tracings. Interobserver agreement ranged from *κ* = 0.65– *κ* = 0.74. Agreement for classification of decelerations was highest (*κ* = 0.79) and lowest for baseline variability (*κ* = 0.50). Overall interobserver agreement was highest for suspicious tracings (*κ* = 0.77) and lowest for normal tracings (*κ* = 0.54).
3.	Palomaki, Luukkaala, Luoto and Tuimala, (2006) Finland [26]	Interobserver variation during the intrapartum period in visual interpretation of CTGs	Quantitative: 22 CTG traces with clinical information but without clinical outcomes of the labor were interpreted by 31 obstetricians.	22 CTG traces of women who had pregnancies complicated by medical disorders or who had caesarian sections.	Fifteen senior obstetricians with more than 4 years’ experience and sixteen junior obstetricians with less than or equal to 4 years’ experience from 10 delivery units.	Agreement- Proportions agreement (Pa) with 95% confidence intervals (CIs)	Normal CTGs: Pa = 0.63–0.82, Power of contractions: Pa = 0.24. Abnormal CTGs: Pa = 0.18–0.60 Clinical decisions: Higher Pa for CTGs without a recommendation for intervention (0.63, 95% CI 0.62–0.64) Intervention recommended: Pa = 0.55 (95% CI 0.54–0.56). Pa for abnormal CTGs was better among senior than among junior obstetricians.
4.	Gyllencreutz, Varli, Lindqvist, Holzmann. (2017) Sweden [27]	Can a combination of web-based CTG education and on-site CTG training lead to a better agreement in CTG interpretation than web-based education alone?	Quantitative multiple-choice questionnaire.	106 CTG tracings	Six obstetricians from two different departments interpreted CTGs on two occasions.	Cohen’s kappa (κ) values for the five parameters assessed on CTGs	Extended on-site CTG training: Higher interobserver reliability for baseline, κ = 0.76, (95% CI = 0.69–0.83) vs κ = 0.71 (95% CI = 0.63–0.78). Variability κ = 0.53 (95% CI = 0.46–0.60) vs κ = 0.41 (95% CI = 0.35–0.48). Accelerations: κ = 0.72 (95% CI = 0.65–0.80) vs κ = 0.60 (0.52–0.68). Similar interobserver agreement for decelerations: κ = 0.42 (95% CI = 0.39–0.46) vs κ = 0.44 (95% CI = 0.41–0.48). Web-based education only: Higher agreement for contractions: κ = 0.69 (95% CI = 0.63–0.74) than κ = 0.58 (95% CI = 0.52–0.63. Extended on-site CTG training, intraobserver reliability was higher for baseline κ = 0.93 (95% CI = 0.90–0.97 vs 0.78 (95% CI = 0.72–0.85), variability κ = 0.80 (95% CI = 0.73–0.86) vs κ = 0.66 (95% CI = 0.58–0.74), accelerations κ = 0.92 (95% CI = 0.87–0.96) vs κ = 0.72 (95% CI = 0.64–0.79), and contractions κ = 0.81 (95% CI = 0.74–0.88) vs κ = 0.74 (95% CI = 0.65–0.84). Similar intraobserver agreement for decelerations κ = 0.65 (95% CI = 0.59–0.70) vs κ = 0.65 (95% CI = 0.60–0.71).
5.	Ghi, Morganelli, Bellussi, Rucci. Giorgetta, Rizzo, Frusca, Pilu, (2016) Italy [31]	The accuracy of the RCOG and the Piquard CTG classification systems in identifying a group of fetuses delivered in the second stage of labor with metabolic acidemia at birth	Quantitative retrospective case–control study.	CTGs from 82 acidemic fetuses and 164 controls.	1 senior consultant (with >10 years of experience in the labor ward and 1 junior trainee with <3 years of experience	Cohen’s Kappa coefficient (κ)	Moderate interobserver agreement, k = 0.58 (95% CI 0.50–0.66) for RCOG, and k = 0.50 (95% CI 0.44–0.56) for Piquard. Pa = 72.8% for RCOG and 60.2% for Piquard classification
6.	Santo, Ayres-De-Campos, Costa-Santos, Schnettler, Ugwumadu, Da Graca, (2016) Portugal, Boston and London [28]	Interobserver agreement, reliability and accuracy of CTG analysis performed according to the FIGO, ACOG and NICE guidelines.	Quantitative	151 tracings of singleton pregnancies, at term, cephalic presentation, absence of known fetal malformations, active labor, continuous CTG indicated	27 clinicians. n = 9 had >10 years’ experience, n = 9 had 6–10 years’ experience, n = 9 had <6 years’ experience	Agreement: Pa Reliability, κ statistic	FIGO Guideline: FHR Baseline: Pa = 0.81 (95% CI = 0.78–0.85) κ = 0.63 (95% CI = 0.57–0.70). Variability: Pa = 0.83 (CI = 0.80–0.86) κ = 0.51 (95% CI = 0.42–0.61). Accelerations: Pa = 0.67 (95% CI = 0.64–0.71) κ = 0.34 (95% CI = 0.29–0.41. Decelerations: Pa = 0.92 (95% CI = 0.89–0.95) κ = 0.53 (95% CI = 0.43–0.66). Classification: Pa = 0.64 (95% CI = 0.61–0.67) κ = 0.37 (95% CI = 0.31–0.43). ACOG Guideline: FHR Baseline: Pa = 0.88 (95% CI = 0.84–0.91) κ = 0.59 (95% CI = 0.49–0.69). Variability: Pa = 0.85 (95% CI = 0.82–0.88) κ = 0.49 (0.39–0.59). Accelerations: Pa = 0.67 (95% CI = 0.63–0.70) κ = 0.34 (95% CI = 0.28–0.40). Decelerations: Pa = 0.85 (95% CI = 0.82–0.88) κ = 0.28 (95% CI = 0.18–0.46). Classification: Pa = 0.73 (95% CI = 0.70–0.76) κ = 0.15 (95% CI = 0.10–0.21). NICE Guideline: FHR Baseline: Pa = 0.88 (95% CI = 0.85–0.91) κ = 0.65 (95% CI = 0.58–0.72). Variability: Pa = 0.83 (95% CI = 0.80–0.86) κ = 0.38 (95% CI = 0.29–0.50). Accelerations: Pa = 0.71 (95% CI = 0.68–0.75) κ = 0.41 (95% CI = 0.35–0.48). Decelerations: Pa = 0.89 (95% CI = 0.85–0.91) κ = 0.47 (95% CI = 0.35–0.59). Classification: Pa = 0.55 (95% CI = 0.51–0.58) κ = 0.33 (95% CI- 0.28–0.39)
7.	Pruksanusak, Thongphanang, Chainarong, Suntharasaj, Kor-anantakul, Suwanrath and Petpichetchian, (2017) Thailand [29]	The agreement of CTG interpretations between the NICHD 3 tier and 5 tier FHR classification systems between physicians with differing levels of experience. To identify the most suitable FHR classification system for use in our clinical practice.	Quantitative prospective study. CTG trace of women who delivered between March and November 2015, labor at term, with a singleton pregnancy	715 independently interpreted by three physicians, blind to anamnestic data	Three physicians, 1 board certified maternal fetal medicine specialist, a second year maternal- fetal medicine fellow, and a 3^rd^ year obstetrics and gynecology chief resident	Cohen’s kappa	Moderate (κ = .52) interobserver agreement for the NIHCD 3-tier system and fair (κ = 0.30) interobserver agreement for the NIHCD 5-tier system. Interobserver agreement for individual CTG parameters was: Baseline (κ = 0.75), variability (κ = 0.01), acceleration (κ = 0.39), early deceleration (κ = 0.37), variable deceleration (κ = 0.59), late deceleration (κ = 0.37), prolonged deceleration (κ = 0.56) Overall intraobserver for NICHD 3 tier classification system was higher (κ = 0.75) than the NIHCD 5 tier system (κ = 0.62). Overall interobserver agreement was higher for the NICHD 3-tier system (κ = 0.52) than the NIHCD 5tier system (0.30)
8.	Amadori, Vaianella, Tosi, Baronchelli, Surico, Remorgida, (2022) Italy [30]	The inter and intra observer agreement for intrapartum CTG interpretation with and without anamnestic data	Quantitative A retrospective interpretation of 73 intrapartum CTGs at time 0 (T0) for a first blind interpretation and at time 1 (T1) two months later with additional anamnestic pregnancy information. Eight different operators	73 CTG traces from deliveries performed in May 2020. The tracings had to be continuous	Four obstetricians and four midwives with different years of work experience	Reliability: Cohen’s kappa coefficient Agreement: κ value of concordance. Average agreement for each profession was measured using the standard deviation.	Moderate concordances among three obstetricians. One CTG: fair agreement, *κ =* 0.39. Substantial agreement among three midwives. One CTG: moderate agreement was moderate, *κ =* 0.58. Moderate interobserver agreement for normal CTGs, no consensus for suspect or pathological CTGs. Clinical data affect the interpretation of suspicious and pathological traces.
9.	Li, Wang, Cai, Zhao, Chen, Liu, Shen, Chen, Li, Zhao, Wang, (2022) China [32]	Agreement and reliability among obstetricians with different skill levels on the interpretation of nonreassuring intrapartum EFM. Accuracy of prediction for neonatal acidemia by obstetricians	Quantitative, retrospective cohort study	100 CTG traces from women who delivered at term with a singleton pregnancy	6 obstetricians with differing years of experience	Agreement- proportional agreement and specific agreement. Reliability- Gwets AC1 Neonatal acidemia- Fisher’s exact test.	Interobserver Pa FHR parameters: baseline FHR (Pa = 0.94; 95% CI = 0.91,0.97), variability (Pa = 0.88), 95% CI = (0.84,0.93), acceleration (Pa = 0.55), 95% CI = (0.52,0.58), early deceleration (Pa = 0.39), 95% CI = (0.36,0.43)), variable deceleration (Pa = 0.57), 95% CI = (0.54,0.62), late deceleration (Pa = 0.77), 95% CI = (0.73,0.82), prolonged deceleration (Pa = 0.70), 95% CI = (0.66,0.75), sinusoidal pattern (Pa = 0.99), 95% CI = (0.98,1.00). Good interobserver agreement for most variables, except early deceleration. Reliability was also prefect among most variables, except for acceleration, early deceleration, and prediction of neonatal acidemia, with low AC1 value 0.17, 0.10, and 0.25, respectively.

Cardiotocograph (CTG), weighted kappa (κw), proportional agreement (Pa); Cohen’s kappa (*κ*); confidence interval (CI); National Institute for Child Health and Human Development (NIHCD); fetal heart rate (FHR), time 0 (T0); time 1 (T1); time 2 (T2); electronic fetal monitoring (EFM).

The advantage of the kappa statistic is that it measures agreement beyond that of chance, which proportion of agreement does not [27].

### Key themes

This review investigated the factors contributing to variation in the visual interpretation of intrapartum cardiotocographs among healthcare professionals. Thematic analysis revealed two themes, namely, interpretational variations among health professionals for cardiotocograph characteristics and factors leading to increased interpretational variation among healthcare professionals. Seven subthemes were also identified. The themes and subthemes are presented in Table 3 below.

**Table 3:** Table of themes and subthemes.

Themes	Sub-Themes
Interpretational variations in cardiotocograph characteristics among health professionals	Cardiotocograph features. Cardiotocograph category classification.
Factors leading to increased interpretational variation among healthcare professionals.	Work experience Professional category Clinical and anamnestic information Cardiotocograph guidelines

#### Theme 1: Interpretational variations in cardiotocograph characteristics among health professionals

The current review identified key areas of interpretational variation among healthcare professionals related to cardiotocograph features.

*Cardiotocograph features*. Agreement of visual interpretations of intrapartum cardiotocographs ranged from fair to good for baseline FHR, baseline variability, accelerations, and frequency of contractions. Interobserver agreement was excellent for the classification of decelerations [3]. Palomaki et al. [26] reported greater proportional agreement for normal features on cardiotocographs than for abnormal features. The interpretation of individual cardiotocograph characteristics using the National Institute for Child Health and Human Development three-tier and 5-tier systems revealed an interobserver agreement of κ = 0.75 for the FHR baseline, κ = 0.01 for baseline variability, κ = 0.39 for accelerations, κ = 0.59 for variable decelerations, κ = 0.37 for late decelerations and κ = 0.66 for prolonged decelerations [29]. For example, a study conducted in China revealed that proportional agreement between participants was good for baseline, variability, acceleration, variability, and late and prolonged decelerations. The feature with the least interobserver agreement was early decelerations (Pa = 0.39) [32]. One study evaluated whether extended cardiotocograph interpretation training improved cardiotocograph interpretation and reported that extended training greatly improves intraobserver agreement for the FHR baseline, variability, accelerations, and contractions. Interobserver agreement was better for the baseline, variability and accelerations [27].

*Cardiotocograph category classification*. The cardiotocograph category classification varied among different healthcare professionals, while intraobserver agreement also varied in some instances. Devane and Lalor [3] revealed that intraobserver agreement for trace classification was altered between time 1 and time 2 for normal traces 18% of the time. Intraobserver variation was also noted for 29% of the time for suspicious traces and 11% of the time for pathological traces. The same study revealed that interobserver agreement was greatest for suspicious traces (κ = 0.77) and lowest for normal traces (κ = 0.54). In contrast, Blix et al [25] reported that interobserver agreement was highest for normal traces (Pa >0.85). A similar result was observed by Palomaki et al. [26], who reported that interobserver agreement was greater for traces that did not require any intervention (Pa = 0.63) than for traces where clinical intervention was recommended (Pa = 0.55). Similarly, Amadori et al. [30] reported moderate interobserver agreement in classifying cardiotocograph traces as normal but reported no consensus on suspicious and pathological traces. Another study that compared the National Institute of Child Health and Human Development (NICHD) three-tier and five-tier classification systems revealed that both classification systems showed strong agreement for normal cardiotocograph traces [29].

#### Theme 2: Factors leading to increased interpretational variation among healthcare professionals

This review revealed several factors linked to variations in the interpretation of intrapartum cardiotocographs, including work experience, professional category, clinical and anamnestic information, and cardiotocography guidelines.

*Work experience*. One study [26] compared interobserver agreement between senior and junior obstetricians and revealed that senior obstetricians demonstrated better agreement when assessing cardiotocograph abnormalities (Pa = 0.59) than did junior obstetricians (Pa = 0.51). In that study, senior doctors were defined as doctors with more than 4 years of work experience, while those with less than 4 years of work experience were categorized as junior doctors. The same study also reported that both senior and junior obstetricians recommended interventions equally. However, junior doctors were 30% more likely to recommend that patients undergo cesarean section. In contrast, another study on interobserver reliability among junior and senior obstetricians reported that interobserver agreement and reliability were not strongly affected by years of experience [32]. That study categorized years of experience into three categories, namely, senior level: having more than 10 years of work experience; intermediate level: having 5–10 years of obstetric experience; and junior level: having less than 3 years of obstetric experience.

*Professional category*. The current review revealed that midwives tend to have more interpretational agreement than other professionals within the multidisciplinary team [30]. That study assessed inter- and intraobserver reliability among midwives and obstetricians. The results of that study found greater intraobserver reliability for midwives (mean agreement % = 77.05%) than for obstetricians (mean agreement % = 65.75%). Midwives were also more likely to have interobserver agreement (κ = 0.58) than obstetricians (κ = 0.39).

*Clinical and anamnestic information*. A study performed in Italy [30] established that a patient’s clinical and anamnestic information reduced obstetricians’ cardiotocograph classification from pathological to suspicious and even to normal. Senior midwives, however, remained relatively consistent in their analysis of cardiotocograph traces with or without clinical and anamnestic data. The same study also revealed that clinical and anamnestic data played a role in the classification of a trace as suspicious or pathological but did not affect the classification of normal traces.

*Cardiotocograph guidelines*. Three doctors with varying levels of expertise compared the NICHD three-tier cardiotocograph classification system and the 5-tier cardiotocograph classification system in Thailand [29]. The same study also compared trace interpretations using FIGO guidelines and an automated computerized system. Overall, the interobserver agreement was fair (κ = 0.52) for the three-tier classification system and moderate (κ = 0.30) for the five-tier classification system. The intraobserver agreement was greater for the NICHD 3-tier system (κ = 0.75) than for the NIHCD 5-tier system (κ = 0.62). Another study performed in Italy investigated the level of interobserver agreement between the Royal College of Gynecologists and the Piquard classification systems [31]. The Piquard classification system is specific to the second stage of labor and describes six different cardiotocograph patterns in relation to fetal pH at birth [37]. The results of that study revealed moderate interobserver agreement for both the RCOG and the Piquard classification systems. A multicountry study investigated interobserver agreement between the FIGO, ACOG, and NICE cardiotocograph classification systems [28]. The results of that multicountry study established that the reliability of the FIGO classification system (κ = 0.37, CI = 0.31–0.43) and the NICE classification system (κ = 0.33, CI = 0.28–0.39) was significantly greater than that of the ACOG classification system (κ = 0.15, CI = 0.10–0.21).

## Discussion

This current review aimed to identify the factors contributing to variations in the interpretation of intrapartum cardiotocographs among healthcare professionals. Evidence suggests that there is marked intra- and interobserver variability in the visual interpretation of intrapartum cardiotocographs. This variability may be attributed to the subjective nature of visual interpretation. The current review also revealed that the best agreement was observed for normal intrapartum cardiotocograph traces. There are conflicting data about whether work experience influences the level of agreement between professionals during interpretation. Anamnestic and clinical data may also alter interpretation, and the cardiotocograph classification guidelines may influence interpretation.

Most of the included studies reported high interobserver agreement for normal traces [25,26,29]. These findings align with those of a systematic review by Englehart et al. [6], who reported greater reliability and agreement between observers for individual cardiotocograph features than for overall cardiotocograph classification. Furthermore, their review reported high interobserver agreement for the classification of normal traces. This is concerning because it is suspicious and pathological traces which warrant interventions during labor.

Studies in this current review revealed more inter- and intraobserver agreement among midwives [30]. Amadori et al. [30] observed that midwives might be more inclined to interpret cardiotocographs accurately because they are present throughout labor, which allows them to watch labor unfolding both for physiological and pathological traces. Determining whether work experience affects cardiotocograph interpretation is difficult due to conflicting results. Thellesen et al. [38] revealed that participants with more than 15–20 years of work experience scored higher on cardiotocograph interpretation than participants with less than 15 years of work experience. The findings of that same study also revealed that the size of the maternity unit affects cardiotocograph interpretation expertise. On the topic of interventions following cardiotocograph interpretation, this current review revealed that junior doctors were most likely to recommend a cesarean section as an intervention following cardiotocograph interpretation.

With regard to the influence of clinical and anamnestic data on cardiotocograph interpretation variation, the NICE [11], FIGO [9], and ACOG [10] guidelines all agree that clinical and anamnestic data should be considered during cardiotocograph interpretation. The three guidelines recommend that cardiotocograph interpretation be performed simultaneously with an ongoing risk assessment of antenatal or intrapartum risks that may affect the fetus and the progress of labor.

## Implications for practice

- Labor wards with junior staff should create opportunities for mentoring relationships between junior and senior staff in the area of cardiotocograph analysis to allow for knowledge sharing and transfer.
- The practice of sending cardiotocographs to obstetricians without any anamnestic or clinical patient data for analysis and clinical decision-making should be critically reviewed.
- Continuous training on cardiotocograph interpretation focusing mainly on suspicious and pathological traces should be offered to all staff who are involved in intrapartum care.
- Low- and middle-income countries need to find innovative solutions to aid midwives and obstetricians who do not have access to adjunct technologies found in high-income countries to aid in the confirmation of fetal distress during labor. Solutions should be cost-effective, guarantee maternal and fetal safety and ensure practical implementation. This will help to achieve accurate cardiotocograph interpretation, which will ultimately reduce the cesarian section rate.

## Limitations and strengths of the review

This review was limited to studies investigating noninvasive cardiotocograph interpretation without adjunctive technologies such as ST segment analysis, fetal blood sampling, and umbilical cord pH analysis. Another limitation is that only studies written in the English language were included. The reviewers conducted a comprehensive search of the included databases and a citation search of all the included full-text articles. However, it is possible that some relevant studies could have been missed. However, the review included all the studies that met the inclusion criteria. Furthermore, a quality appraisal of all included studies was performed using the QAREL checklist.

## Conclusion

This integrative review investigated factors contributing to variability in the visual interpretation of intrapartum cardiotocographs among healthcare professionals. The results suggest that there is agreement in the interpretation of cardiotocograph features; however, discrepancies are noted when professionals need to classify traces. The work experience of clinicians may affect the agreement of cardiotocograph interpretation. Patient clinical and anamnestic data also guide healthcare professionals in placing cardiotocographs into appropriate categories. Guidelines for classifying cardiotocographs also affected the interpretational agreement between healthcare professionals. Although our review revealed conflicting evidence, more efforts need to be made to develop methods for verifying cardiotocograph results that can also be applied in resource-limited settings with a high incidence of HIV. The training of midwives and obstetricians on visual interpretation should also focus on the interpretation of suspicious and pathological traces.
